# Pheochromocytoma‐Induced Reversible Dilated Cardiomyopathy: A Case Report and Literature Review

**DOI:** 10.1002/ccr3.70241

**Published:** 2025-03-07

**Authors:** Fatemeh Afra, Mohammad Rahimi, Mahboobeh Hemmatabadi, Somayeh Mohammadi, Nooshin Shirzad

**Affiliations:** ^1^ Sina Hospital Tehran University of Medical Sciences (TUMS) Tehran Iran; ^2^ Department of Endocrinology, Vali‐Asr Hospital, Endocrinology and Metabolism Research Center, Imam Khomeini Complex Hospital Tehran University of Medical Sciences Tehran Iran; ^3^ Department of Cardiology, Imam Khomeini Complex Hospital Tehran University of Medical Sciences Tehran Iran; ^4^ Endocrinology and Metabolism Research Center, Endocrinology and Metabolism Clinical Sciences Institute Tehran University of Medical Sciences Tehran Iran

**Keywords:** adrenalectomy, cardiomyopathy, catecholamines, hypertension, pheochromocytoma

## Abstract

Pheochromocytoma is a rare, primarily benign tumor that arises from the chromaffin cells of the adrenal medulla. It is known for causing excessive secretion of catecholamines, such as epinephrine and norepinephrine. This condition poses significant health risks due to its potential to cause severe cardiovascular complications, including hypertension, stroke, and various forms of cardiomyopathy. A 29‐year‐old woman with a history of diabetes mellitus (DM) and hypertension presented with symptoms suggestive of an infection related to an intrauterine device (IUD). However, persistent symptoms following the removal of the IUD and further diagnostic evaluations revealed dilated cardiomyopathy (DCM) secondary to catecholamine excess, which led to the diagnosis of pheochromocytoma. This diagnosis was confirmed through comprehensive laboratory testing and imaging. The surgical removal of the tumor resulted in significant improvement in her cardiac function and the resolution of both hypertension and DM. This case highlights the importance of considering pheochromocytoma in the differential diagnosis of unexplained cardiac symptoms, particularly in young patients with hypertension. The varied clinical presentation can complicate the diagnosis; however, early detection and appropriate management, including surgery, can lead to complete recovery. This case also underscores the potentially reversible nature of catecholamine‐induced cardiomyopathy when the underlying tumor is treated effectively.

**Summary**

- Consideration of pheochromocytoma as a potential cause of dilated cardiomyopathy in young individuals.

## 1 Introduction

Pheochromocytoma, a primarily benign and rare tumor, originates from the chromaffin cells within the adrenal medulla. It is known for causing excessive secretion of catecholamines, such as epinephrine and norepinephrine [1]. The symptoms of this condition are notably variable and include tachycardia, episodic headaches, and sweating, which complicate timely diagnosis.

The most common medical complication from pheochromocytoma is sustained or episodic hypertension, although the range of potential complications is broad [2]. The major health risks associated with pheochromocytoma stem from the overproduction of catecholamines, leading to severe hypertension, stroke, cardiac arrhythmias, myocardial infarction, and even acute heart failure. These severe cardiovascular effects underscore the urgent need for early detection and treatment of the condition [3, 4, 5].

Cardiomyopathy, although a rarer complication, represents a significant cardiac issue characterized by abnormalities in the heart muscle [5]. Research indicates that the type of cardiomyopathy resulting from pheochromocytoma varies with the duration of catecholamine exposure [6]. Common forms include dilated cardiomyopathy (DCM), takotsubo cardiomyopathy (TCM), and hypertrophic cardiomyopathy (HCM) [7].

Extended exposure to high catecholamine levels in individuals with untreated pheochromocytoma directly damages the heart muscle, leading to inflammation and potentially cardiomyopathy. This damage is exacerbated as norepinephrine concentrations increase, driven by β‐adrenergic receptor stimulation, which elevates cAMP and calcium influx, thereby reducing cardiomyocyte viability [5, 8].

The definitive treatment for pheochromocytoma is the surgical removal of the tumor, which often significantly improves symptoms and resolves cardiac complications [9].

In this case report, we discuss a young woman who experienced reversible pheochromocytoma‐induced dilated cardiomyopathy (DCM) following surgical intervention.

## 2 Case History/Examination

A 29‐year‐old Persian/Caucasian woman (weight = 73 kg, height = 165 cm, BMI = 26.81 kg/m^2^) with a history of diabetes mellitus (DM) and hypertension, both diagnosed 1 year ago, and no family history of endocrine or metabolic disorders such as pheochromocytoma or multiple endocrine neoplasia (MEN) subtypes (except for type 2 diabetes in her mother), presented to our hospital with symptoms of cough, abdominal pain, and fever (temperature = 38.5°C, oxygen saturation = 96%, respiratory rate = 18 per minutes, pulse rate = 98 beats per minutes, blood sugar (BS) = 180 mg/dL, and blood pressure (BP) = 100/78 mmHg). Upon presentation, the patient exhibited blood pressure fluctuations, with crises exceeding 180/100 mmHg that decreased to 140/78 mmHg with antihypertensive medication. Her medication history included Metformin 1000 mg, Gliclazide 60 mg, Linagliptin 5 mg, Empagliflozin 10 mg, and Losartan 25 mg tablets.

In the month preceding hospitalization, the patient had multiple doctor visits due to recurrent fever and chills and was treated with antibiotics and nonsteroidal anti‐inflammatory drugs (NSAIDs).

Given her use of an intrauterine device (IUD), an intrauterine infection was suspected, leading to the removal of the IUD; however, her symptoms persisted. She also reported palpitations, sweating, headaches, and a significant weight loss of eight kilograms over the past 2 months.

## 3 Differential Diagnosis, Investigations, and Treatment

**FIGURE 1:**
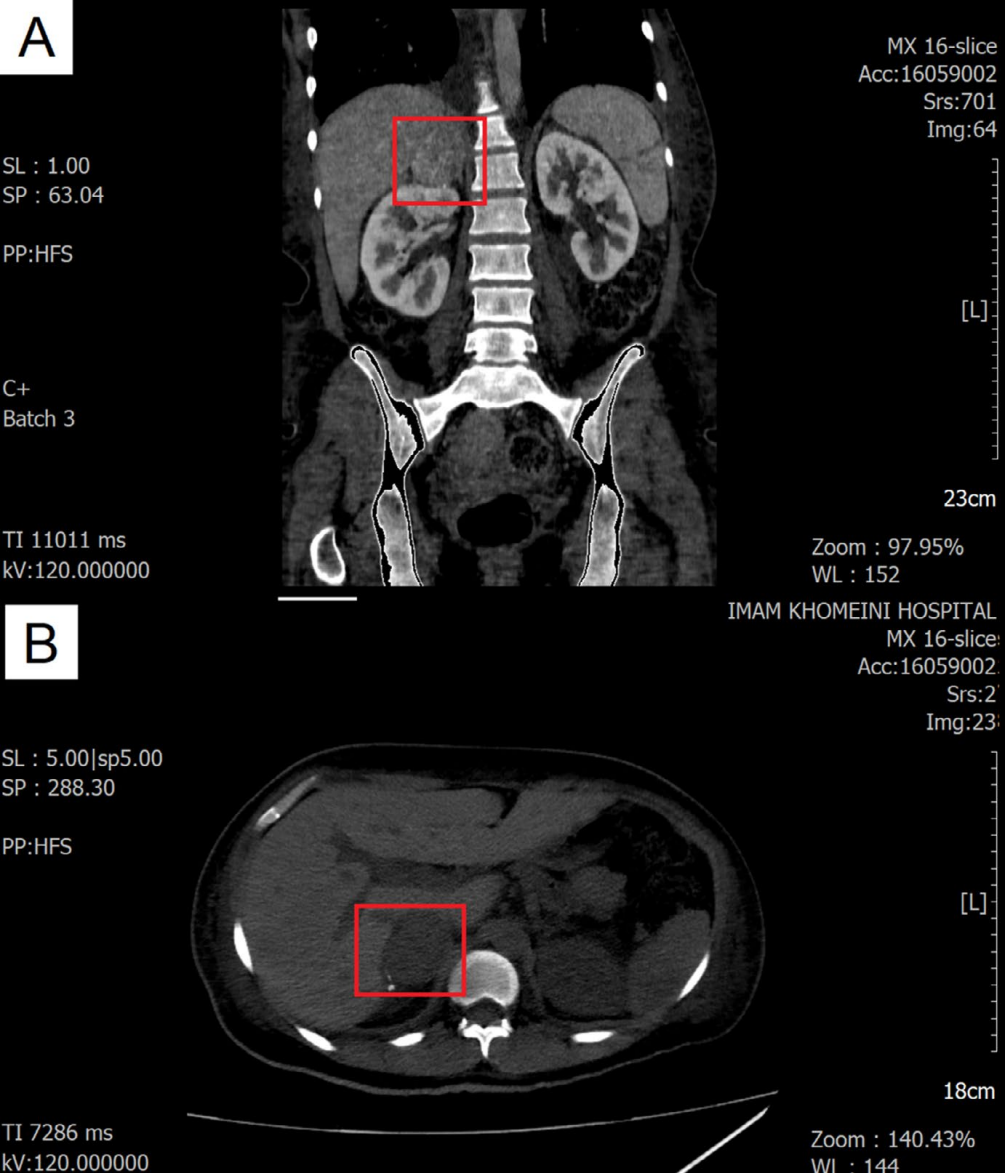
Right adrenal mass. The red square indicates the tumor area. Non‐contrast CT scan showing a Hounsfield unit of 36. After 2 min, the relative and absolute washout rates are 26% and 47%, respectively.

A blood sample was drawn, and her laboratory findings are summarized in Table 1. Based on her symptoms, laboratory results, and the presence of an S3 sound on auscultation, an electrocardiogram (ECG) and transthoracic echocardiography (TTE) were performed. Moderate left ventricular (LV) enlargement and severe LV dysfunction were observed. Additionally, moderate right ventricular (RV) enlargement and dysfunction were noted. Severe tricuspid regurgitation (TR) and mild to moderate mitral regurgitation (MR) were also present. The pulmonary artery pressure (PAP) was 45 mmHg, and the tricuspid regurgitation gradient (TRG) was 35 mmHg. The left ventricular ejection fraction (LVEF) was estimated to be 10%–15%, and Bisoprolol 2.5 mg twice daily was added to her medications because of her symptoms. For further investigation, cardiac magnetic resonance imaging (MRI) was performed. The findings showed global hypokinesia in the left LV, with an enlarged LV size, no LV hypertrophy, and a severely reduced LVEF of 14%. Additionally, the RV was of normal size, without RV hypertrophy, but with a severely reduced RV EF of 20%. This revealed sinus tachycardia on the ECG, and the echocardiogram indicated heart failure, possibly related to cardiomyopathy. Based on the computed tomography (CT) scan findings (Figure 1) and urinary assessments for vanillylmandelic acid (VMA), metanephrine, and normetanephrine (Table 1), a diagnosis of pheochromocytoma was confirmed. Additionally, given the elevated NT‐proBNP levels and other cardiac symptoms, along with insights from previous case studies [1, 4, 10, 11, 12, 13], our suspicion was directed toward pheochromocytoma‐induced cardiomyopathy.

**TABLE 1:** Laboratory findings at admission.

Variable	Result	Reference range	Variable	Result	Reference range	Variable	Result	Reference range
WBC (×1000 mm^3^)	14.5	4.0–10.0	ALT (U/L)	26	< 31	Alb (g/dL)	3.6	3.5–5.2
Neutrophils (%)	68.3	—	AST (U/L)	20	< 31	Troponin‐I (μg/L)	< 0.20	Up to 0.29
Lymphocytes (%)	27.4	—	ALP (U/L)	120	70–306	NT‐proBNP (pg/mL)	2600	Up to 125
Hemoglobin (g/dL)	13.4	12.0–16.0	HbA1c (%)	8.4	< 5.6	Anti‐GAD (IU/mL)	0.5	< 10.0
MCV (fL)	74	80–100	C‐peptide (ng/mL)	3.12	1.0–4.8	Renin (AU/mL)	3.0	5.3–99.1
PLT (×1000mm^3^)	354	150–450	Total cholesterol (mg/dL)	109	< 200	Aldosterone (pg/mL)	44.8	14.6–324
Na (meq/L)	145	135–145	TG (mg/dL)	93	< 150	Urine analysis	Negative for bacteria and yeast	—
K (meq/L)	5.0	3.5–5.0	HDL (mg/dL)	34	> 35	Urine/blood culture	Negative	—
Ca (mg/dL)	9.3	8.6–10.2	LDL (mg/dL)	56	< 100	Urine volume (mL/24 h)	2300	600–2400
P (mg/dL)	5.1	2.5–5.0	CRP (mg/L)	5.0	< 6.0	Urine creatinine volume (mg/24 h)	253	740–1570
Urea (mg/dL)	23	15–50	ESR (mm/h)	9	Up to 20	Urine VMA (mL/24 h) (HPLC) (mg/24 h)	1.3	Up to 13.6
Creatinine (mg/dL)	0.6	0.7–1.4	TSH (mIU/mL)	1.4	0.3–4.7	Urine normetanephrine (μg/24 h) (ELISA)	2400	Up to 600
Bil T	0.8	0.1–1.2	FT4 (pmol/L)	17	10.0–22.0	Urine metanephrine (μg/24 h) (ELISA) (mcg/24 h)	118.9	Up to 350
Bil D (mg/dL)	0.8/0.4	< 0.3	FT3 (pmol/L)	4.1	3.5–7.0			

Abbreviations: Alb, albumin; ALP, alkaline phosphatase; ALT, alanine transaminase; Anti‐GAD, anti‐glutamic acid decarboxylase antibody; AST, aspartate transaminase; Bil T/D, bilirubin total/direct; CRP, C‐reactive protein; ESR, erythrocyte sedimentation rate; HbA1c, hemoglobin A1c; HDL, high‐density lipoprotein; LDL, low‐density lipoprotein; MCV, mean corpuscular volume; NL, normal range; NT‐proBNP, N‐terminal pro b‐type natriuretic peptide; PLT, platelet; TG, triglycerides; WBC, white blood cell.

**FIGURE 2:**
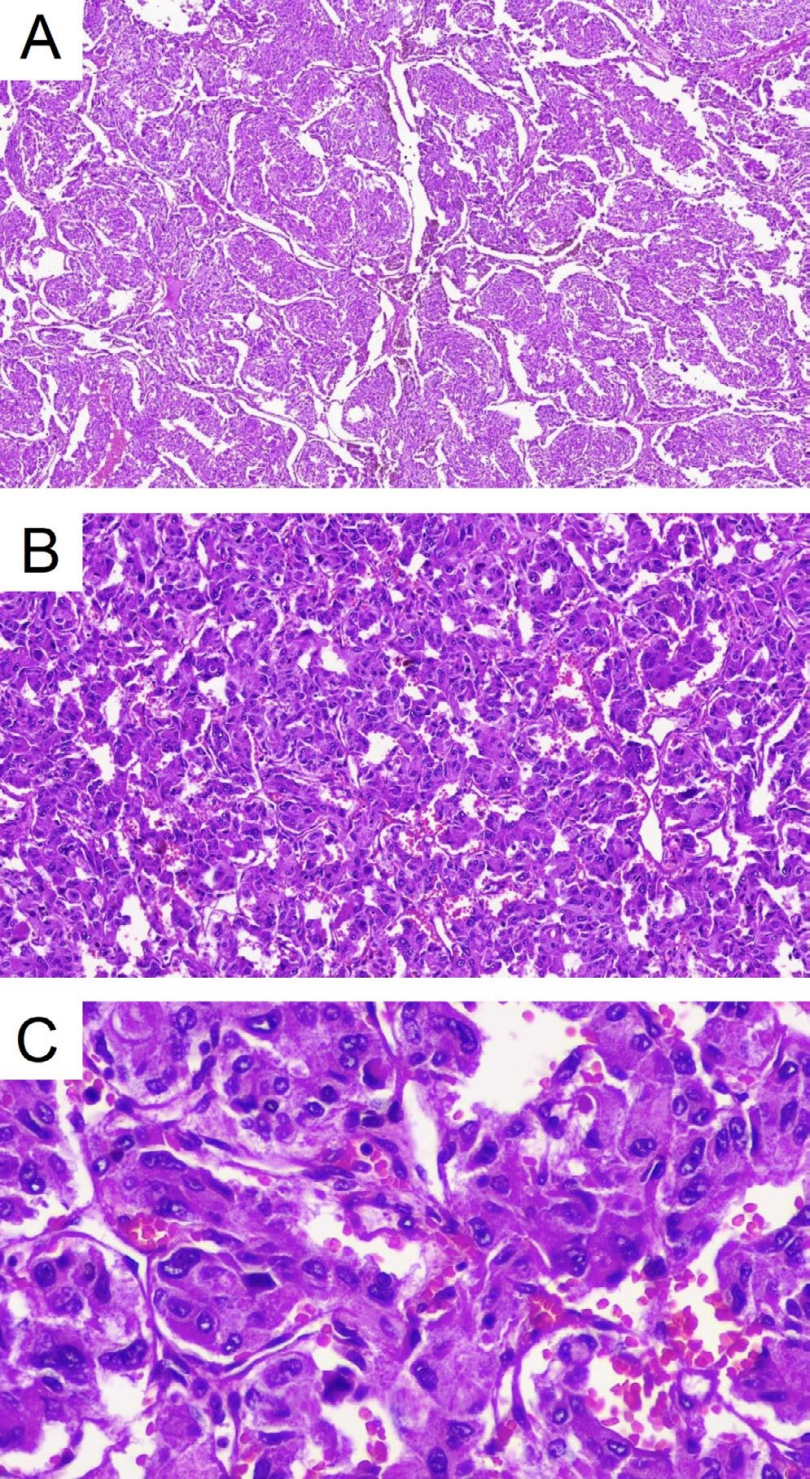
Polygonal or spindle cells arranged in small nests (Zellballen pattern). A, B, and C are at 4×, 10×, and 40× magnification, respectively.

Following the confirmation of the diagnosis, treatment for the hypertensive crisis and other complications was begun. Phenoxybenzamine (10 mg, daily) was added to her medication regimen, and the Bisoprolol dose was increased to 5 mg twice daily to manage her elevated heart rate. Additionally, the patient was scheduled for an adrenalectomy. After the laparoscopic adrenalectomy, she was discharged with the following medications: Spironolactone (25 mg, daily), Ivabradine (2.5 mg, twice daily), Bisoprolol (2.5 mg, daily), Furosemide (20 mg, daily), Vitamin B1 (300 mg, daily), Folic acid (1 mg, daily), Sacubitril/Valsartan (200 mg, twice daily), and Metformin (500 mg, twice daily). Pathology confirmed the presence of a pheochromocytoma exhibiting a Zellballen pattern, with a Ki‐67 index of 1%. Vascular and capsular invasion were present, but comedonecrosis was not identified. The cellularity was moderate. In the ancillary study, synaptophysin and chromogranin were strongly positive in the tumor cells. Total CK AE1/AE3 and PAX8 tested negative, while S100 was positive in the sustentacular cells (Figure 2).

## 4 Conclusion and Results (Outcome and Follow‐Up)

Postoperatively, her symptoms improved, and notably, 10 days after the surgery, the laboratory findings returned to the normal range (Table 2). One month after the surgery, her fasting blood sugar (FBS) level and hemoglobin A1c (HbA1c) were within the normal range. Diabetes mellitus following pheochromocytoma occurs due to increased insulin resistance and reduced insulin secretion caused by excessive catecholamine release. Therefore, tumor resection often leads to significant improvements in glycemic control. The HbA1c level decreased to 6.0%, and blood pressure also decreased. Additionally, her dilated cardiomyopathy (DCM) completely recovered approximately 3 months post‐surgery, with her left ventricular ejection fraction (LVEF) increasing to 55%. Follow‐up echocardiography showed that both the left and right ventricular sizes were normal, with no significant valvular heart disease (VHD) observed. After 2 months, her HbA1c levels improved, and her cardiovascular symptoms subsided. All her medications were discontinued; however, the patient was advised to undergo long‐term follow‐up.

**TABLE 2:** Laboratory findings and urine analysis results 10 days after surgery.

Variable	Result	Reference range	Variable	Result	Reference range
WBC (×1000mm^3^)	9.1	4.0–10.0	Creatinine (mg/dL)	0.9	0.7–1.4
Neutrophils (%)	88.1	—	HbA1c (%)	7.9	< 5.6
Lymphocytes (%)	10.6	—	Ca (mg/dL)	9.2	8.6–10.2
Hemoglobin (g/dL)	9.6	12.0–16.0	P (mg/dL)	3.0	2.5–5.0
MCV (fl)	73.8	80–100	Urine volume (mL/24 h)	2800	600–2400
PLT (×1000mm^3^)	339	150–450	Urine creatinine volume (mg/24 h)	897	740–1570
Na (meq/L)	143	135–145	Urine VMA (mL/24 h) (Chromatography)	6.5	Up to 13.6
K (meq/L)	3.8	3.5–5.0	Urine Metanephrine (μg/24 h) (ELISA)	255	Up to 600
Urea (mg/dL)	39	15–50	Urine Normetanephrine (μg/24 h) (ELISA)	372	Up to 350
NT‐proBNP (pg/mL)	743	Up to 125			

Abbreviations: HbA1c, hemoglobin A1c; MCV, mean corpuscular volume; NL, normal range; NT‐proBNP, N‐terminal pro b‐type natriuretic peptide; PLT, platelet; VMA, vanillylmandelic acid; WBC, white blood cell.

### 4.1 Search Strategy for Literature Review

A literature search was conducted on PubMed using the syntax “Pheochromocytoma‐Induced Cardiomyopathy”[Title/Abstract] AND (Pheochromocytoma [Title/Abstract] OR Cardiomyopathy [Title/Abstract] OR Catecholamines [Title/Abstract] OR Adrenalectomy [Title/Abstract] OR Hypertension [Title/Abstract]) and keywords including “pheochromocytoma AND dilated cardiomyopathy.” All relevant articles were retrieved and their references were manually reviewed to identify additional related articles. (Table 3).

**TABLE 3:** Demographics, clinical features, definitive treatment, and outcomes of cases that reported pheochromocytoma‐induced dilated cardiomyopathy.

Author	Age/gender	Experienced signs/symptoms	Diagnosis method(s)	Echocardiography before management	Procedure	Outcome
Boro et al. [10]	38/male	New onset diabetes mellitus, abdominal pain, episodes of palpitation, anxiety, severe headache, sweating, hypertension, dyspnea, orthopnea, mild swelling in both ankles	CT scan, elevated urine metanephrine and normetanephrine	Dilated LA and LV with concentric LV hypertrophy and global LV hypokinesia, LVEF = 40%	Surgery (right adrenalectomy)	Completely recovered 1 month after surgery
Brilakis et al. [14]	43/female	Sever substernal chest pain, dyspnea, diaphoresis, nausea, vomiting, hypertension, tachycardia	CT scan, elevated urine metanephrine	Generalized hypokinesis, LVEF = 30%	Surgery (left adrenalectomy)	Recovered
Dalby et al. [15]	10/male	Lethargy, malaise, occasional night sweats, heart failure, headache,^a^ palpitation,^a^ hypertension^a^	CT scan, elevated urine VMA	Large ventricle with very poor global contraction, LVEF = 26%	Surgery (right adrenalectomy)	Died because of transplant‐related CAD 64 months post‐surgery
Dalby et al. [15]	48/male	Dyspnea, weight loss, pulmonary edema, palpitation, hypertension,^a^ sinus tachycardia^a^	CT scan, elevated urine noradrenaline	Large, poorly contracting left ventricle, LVEF = 37%, mild mitral regurgitation	Surgery (right adrenalectomy)	Completely recovered
Feng et al. [16]	19/male	Tachypnea, palpitation, legs edema, hypertension, sinus tachycardia, crackles of pulmonary RLL	CT scan	Large LV, diffusive contractive dysfunction for LV, LVEF = 25%	Surgery (left adrenalectomy)	Recovered
Gursoy et al. [17]	25/female	Dyspnea, orthopnea, palpitation, fatigue, hypertension, chest pain, legs edema, sinus tachycardia, tachypnea, diabetes mellitus	MRI, Elevated urine VMA, metanephrine, epinephrine, and norepinephrine	Dilated LV, LV hypokinesia, LVEF = 12%	Surgery (right adrenalectomy)	Recovered
Hamada et al. [11]	54/male	Nausea, vomiting, cold sweating, palpitation, oliguria, hypertension, chest pain, abdominal pain, fever	CT scan, elevated plasma noradrenaline and adrenaline, elevated urine VMA	—	Surgery (right adrenalectomy)	Recovered 2 years post‐surgery
Hicks et al. [12]	15/female	Sweating, lethargy, shortness of breath, palpitation, abdominal pain, pulmonary edema	CT scan, elevated urine norepinephrine and dopamine	LV global hypokinesia, LVEF = 34%	Surgery (right adrenalectomy)	Completely recovered during 6 months
Imperato‐McGinley et al. [13]	12/female	Anorexia, weight loss, nausea, vomiting, abdominal pain, sweating, hypertension, tachycardia	CT scan, elevated plasma norepinephrine, elevated urine metanephrine and VMA	LVEF = 27%	Surgery (right adrenalectomy)	Recovered
Kanemoto et al. [18]	57/male	Palpitation, high blood glucose	CT scan, elevated plasma epinephrine, and norepinephrine Elevated urine epinephrine, norepinephrine, and VMA	LA enlargement, RV and LV enlargement, intra ventricular septum and LV posterior wall hypokinesia, LVEF = 35%	Pharmacotherapy	Died
Kelley et al. [19]	50/male	Paroxysmal AF, malaise, shortness of breath, palpitation. Orthopnea, exertional dyspnea, lower extremity edema	CT scan, elevated urine metanephrine and normetanephrine	Mitral valve regurgitation, LVEF = 25%	Surgery (right adrenalectomy)	Recovered
Lin et al. [5]	65/male	Exertional dyspnea, orthopnea, hypertension, anxiety, dizziness, paroxysmal sweating	CT scan, elevated urine VMA	Diffuse LV hypokinesia, dilation, LVEF = 35%	Surgery (left adrenalectomy)	Recovered during 4‐month period
Lyu et al. [20]	54/female	Palpitation, chest pain, sweating, hypertension, tachycardia, tachypnea	CT scan, elevated serum metanephrine	LVEF = 20%	Surgery (right adrenalectomy)	Recovered
McEntee et al. [21]	43/female	Chest pain, nausea, vomiting, shortness of breast, palpitation, diaphoresis, episodic headache, hypertension	CT scan, elevated plasma normetanephrine, Elevated urine normetanephrine and total metanephrines	LV dilation, diffused LV hypokinesia, LVEF = 25%–30%	Surgery (right adrenalectomy)	Recovered
Molaei et al. [1]	7/male	Malaise, abdominal pain, polydipsia, myalgia, tachycardia, diabetes mellitus, hypertension, nausea, sudoresis	MRI, MIBG scan, elevated urine norepinephrine and normetanephrine	Mild pericardial effusion, mild LV hypertrophy, LVEF = 30%–35%	Surgery (left adrenalectomy)	Recovered
Mootha et al. [22]	34/female	Palpitation, lightheadedness, chest tightness	MRI, MIBG scan, elevated urine epinephrine and metanephrine	LVEF = 20%	Surgery (right adrenalectomy)	Recovered
Nanda et al. [23]	18/pregnant female	Nausea, vomiting, abdominal cramps, hypertension, sinus tachycardia, cyanosis	CT scan, MRI	Severe wall motion akinesia, LVEF = 15%	Surgery (retroperitoneal lesion resection)	Recovered
Sadowski et al. [24]	35/female	Diaphoresis, hot flushes, hypertension, sinus tachycardia	CT scan, elevated urine VMA, metanephrine and catecholamine	Moderate LV dilation moderate LV hypertrophy, severe global hypokinesia, LVEF = 30%	Surgery (left adrenalectomy)	Recovered
Satendra et al. [4]	40/male	Nonproductive cough, weight loss, anorexia, diaphoresis, hypertension, tachycardia, orthopnea	CT scan, elevated urine normetanephrine and epinephrine	—	Surgery (left adrenalectomy)	Recovered
Varghese et al. [25]	42/male	Palpitation, headache, sweating, hypertension, tachycardia	US, MIBG scan, elevated urine metanephrine and normetanephrine	Global hypokinesia, LVEF = 31%	Surgery (right adrenalectomy)	Recovered
Zörner et al. [26]	33/male	Sudden headache, chest pain, hypertension, tachycardia, tachypnea	CT scan	LV lateral hyperkinesia, LVEF = 20%–30%	Surgery (left adrenalectomy)	Recovered

Abbreviations: AF, atrial fibrillation; CAD, coronary artery disease; CT scan, computed tomography scan; LA, left atrium; LV, left ventricle; LVEF, left ventricular ejection fraction; MIBG, iodine meta‐iodobenzylguanidine; MRI, magnetic resonance imaging; RLL, right lower lobe; US, ultra sonography; VMA, vanillylmandelic acid.

^a^ After heart transplantation.

## 5 Discussion

We report a 29‐year‐old woman with DCM who experienced complete recovery following adrenalectomy.

Pheochromocytoma, a rare tumor type, occurs in less than 0.2% of patients with hypertension, with an annual incidence of approximately 0.8 cases per 100,000 person‐years [27, 28, 29]. This condition arises primarily in the adrenal glands or sympathetic ganglia (paragangliomas), which produce hormones like adrenaline and noradrenaline, leading to various complications and symptoms [10, 30]. Pheochromocytomas may manifest either as sporadic instances or as components of hereditary syndromes, such as Multiple Endocrine Neoplasia type‐2 (MEN‐2), Von Hippel–Lindau syndrome (VHL), and Neurofibromatosis type‐1 (NF‐1), depending on their prevalence and frequency of occurrence [31, 32, 33]. Although the clinical presentation of pheochromocytoma varies with the location of the tumor and the amount of catecholamine secretion, common symptoms include the classic triad of palpitations, sweating, and headaches [30]. One of the rare complications of pheochromocytoma is cardiomyopathy, which results from prolonged catecholamine exposure. Chronic exposure to catecholamines increases the inotropic and chronotropic effects on the heart through overactivation of beta‐1 receptors, consequently elevating the oxygen demand of the myocardium and inducing hypoxia. This leads to heightened oxidative stress and increased permeability of the sarcolemma to calcium influx, resulting in inflammation, interstitial fibrosis, and necrosis [17]. Ultimately, these processes cause the ventricles to become overworked, potentially leading to heart failure and myocardial apoptosis. Additionally, catecholamine binding to postsynaptic alpha‐1 receptors causes vasoconstriction of blood vessels and decreases coronary blood flow [10, 34]. Furthermore, excessive catecholamines increase pulmonary capillary pressure, leading to congestive symptoms. Together, these mechanisms activate a negative feedback pathway in beta‐1 adrenergic receptors, which decreases cardiac function and produces symptoms that mimic heart failure, thereby complicating the differential diagnosis [17, 35]. In a comprehensive review of case reports on pheochromocytoma‐induced dilated cardiomyopathy (DCM), we observed that patients present with a variety of signs and symptoms (Table 3). Most do not exhibit the classic pheochromocytoma triad; however, hypertension and palpitations are among the most commonly reported symptoms [36]. Variations in the amount and types of catecholamine metabolites excreted in urine or plasma are also evident, reflecting differences in the severity of patients' conditions and genetic variability in catecholamine‐degrading enzymes [36]. Additionally, Hamada and colleagues [11] as well as Molaei and co‐authors [1], reported cases where an increase in white blood cells was observed alongside typical symptoms, similar to those in our case. This response is justified within the context of this disease [37].

Early diagnosis, based on appropriate measures before other interventions and considering the episodic nature of symptoms like hypertension and tachycardia, can lead to complete recovery. Moreover, echocardiography, CT scans, and laboratory analyses of secreted catecholamine metabolites in urine or plasma are highly accurate in patients suspected of having pheochromocytoma. It has been reported that the time for complete recovery can vary from a few days to several months post‐surgery, depending on individual circumstances and underlying diseases [11, 13, 17, 22]. However, the possibility of disease recurrence underscores the importance of regular patient follow‐up [23].

The definitive treatment for pheochromocytoma is surgical intervention, the absence of which can be fatal [18]. Prior to surgery, it is crucial to manage blood pressure and other symptoms with medication [34].

Proper preoperative management with alpha‐blockers is essential to reduce hypertensive crises. It is important to note that beta‐blockers should not be administered before alpha‐blockers, such as phenoxybenzamine, as this oversight can lead to a hypertensive crisis and pulmonary edema due to vasoconstriction [10]. Additionally, timely diagnosis and treatment are vital, as delays can result in incomplete recovery or even death [15, 34, 38]. A case remarkably similar to ours was reported by Gursoy and colleagues [17], in which the patient presented with hypertension, the classic symptom triad of headache, tachycardia, and diaphoresis, as well as diabetes mellitus (DM) and a left ventricular ejection fraction (LVEF) of 12%. The patient was found to have a mass in the right adrenal gland. Remarkably, all symptoms, including hyperglycemia, improved shortly after surgery. Molaei and colleagues [1] also reported complete recovery after surgery in a patient presenting with lethargy, abdominal pain, and hyperglycemia.

It is worth noting that considering the evidence of anti‐GAD antibodies, C‐peptide levels, and a history of diabetes in the patient's mother, it appears that the patient's diabetes is type 2, which has been exacerbated by pheochromocytoma. After surgery, the patient's blood glucose levels have been controlled; however, given the HbA1c level of 6, they remain within the prediabetes range.

One limitation we encountered was the patient's refusal to undergo genetic testing due to its high cost. Despite guidelines recommending genetic testing [10] and the potential genetic nature of the disease [1], testing was not performed in this patient. Given that pheochromocytoma patients can present with a variety of clinical and laboratory symptoms, making a differential diagnosis of cardiomyopathy induced by pheochromocytoma—a relatively rare disease—can be challenging. Therefore, pheochromocytoma should be considered as a differential diagnosis, especially in young patients with cardiac symptoms.

## 6 Conclusion

This case report underscores the importance of considering pheochromocytoma in the differential diagnosis of unexplained cardiac symptoms, especially in young patients. We detail the successful identification and surgical treatment of pheochromocytoma in a 29‐year‐old woman diagnosed with dilated cardiomyopathy (DCM) due to catecholamine excess. Her recovery, marked by improved cardiac function and resolution of hypertension and diabetes, highlights the critical role of comprehensive diagnostics and timely intervention.

## Author Contributions

**Fatemeh Afra:** formal analysis, investigation, methodology, resources, visualization, writing – original draft, writing – review and editing. **Mohammad Rahimi:** conceptualization, data curation, investigation, supervision, writing – review and editing. **Mahboobeh Hemmatabadi:** conceptualization, methodology, project administration, supervision, validation, writing – review and editing. **Somayeh Mohammadi:** validation, writing – review and editing. **Nooshin Shirzad:** conceptualization, methodology, project administration, supervision, validation, writing – review and editing.

## Ethics Statement

This case report was composed following the acquisition of the patient's informed consent for its publication. No extra diagnostic tests or treatment strategies were conducted specifically for this case report.

## Consent

The patient provided written informed consent for the publication of this case report and any related images. The written consent document is available for the Editor‐in‐Chief of this journal to review upon request.

## Conflicts of Interest

The authors declare no conflicts of interest.

## Data Availability

Data available on request from the authors.
